# Current approaches to predicting intraventricular hemorrhage in preterm neonates using blood biomarkers

**DOI:** 10.3389/fped.2025.1706119

**Published:** 2025-11-28

**Authors:** Marie Kasíková, Pavel Brož, Jiří Dort, Ondřej Topolčan

**Affiliations:** 1 Department of Neonatology, University Hospital in Pilsen, Pilsen, Czechia; 2 Faculty of Medicine in Pilsen, Charles University, Pilsen, Czechia; 3 Institute of Clinical Biochemistry and Hematology, University Hospital in Pilsen, Pilsen, Czechia; 4 Department of Immunochemistry Diagnostics, University Hospital in Pilsen, Pilsen, Czechia

**Keywords:** biomarkers, cerebral intraventricular hemorrhage, extremely preterm infants, early detection of disease, neonate

## Abstract

Intraventricular hemorrhage (IVH) is a serious complication of preterm birth that can lead to permanent neurodevelopmental impairment. In recent years, there has been increasing interest in identifying and validating biomarkers capable of predicting the onset and progression of IVH, as well as other forms of neonatal brain injury. The implementation of reliable biomarkers into routine clinical practice represents a crucial step toward developing effective neuroprotective strategies and preventing irreversible brain damage in preterm infants. This review summarizes the current state of knowledge regarding blood-based biomarkers and their potential to predict the risk of IVH in preterm neonates. We focus on markers of brain injury, inflammatory markers, growth factors, vasoactive substances, indicators of oxidative stress, and emerging findings from the fields of genomics, proteomics, and metabolomics. We highlight the most promising biomarker candidates, discuss their biological plausibility, and critically evaluate the methodological challenges currently limiting their clinical translation. Furthermore, we outline future research directions that may facilitate the development of reliable diagnostic tools and promote the integration of personalized neuroprotective strategies into neonatal care. The early and accurate prediction of IVH remains a major unmet need in neonatology, and advances in biomarker-based approaches hold considerable promise for guiding clinical decision-making and improving long-term neurodevelopmental outcomes in preterm infants.

## 1 Introduction

### 1.1 Intraventricular hemorrhage

In the care of preterm infants, clinicians constantly face the risk of numerous serious complications associated with prematurity, particularly in extremely preterm infants. One of the most severe complications is intraventricular hemorrhage (IVH), which can result in profound neurodevelopmental impairment (1–5). Despite ongoing advances in neonatal intensive care, the global incidence of IVH in preterm infants remains high, ranging from 15% to 45%. This variability is influenced by gestational age, regional disparities in healthcare, and differences in neonatal intensive care unit protocols. Importantly, the incidence of IVH increases as gestational age at birth decreases (6).

**Figure 1:**
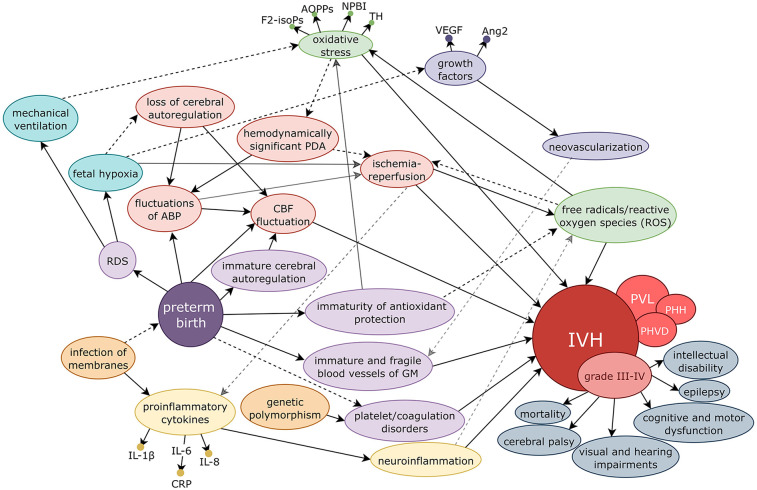
Schematic representation of the multifactorial pathogenesis of IVH and related complications in preterm neonates. Intrauterine infection and inflammation contribute to preterm birth and are associated with the release of proinflammatory cytokines, which promote neuroinflammation and endothelial dysfunction. Preterm neonates are further predisposed to hemorrhage due to the immaturity of the GM vasculature, insufficient antioxidant defenses, and impaired cerebral autoregulation. Fetal hypoxia, commonly secondary to RDS, hemodynamically significant PDA, or systemic hypotension, leads to fluctuations in ABP and CBF. These fluctuations, along with ischemia–reperfusion injury, exacerbate oxidative stress and the production of ROS. Simultaneously, hypoxia induces the expression of angiogenic growth factors (VEGF, Ang2), resulting in fragile and immature neovascularization within the GM. The combined effects of vascular fragility, inflammation, oxidative stress, and hemodynamic instability culminate in GM hemorrhage and subsequent IVH. Severe IVH (grade III–IV) is associated with acute complications, including PHVD, PHH, and PVL. Adverse outcomes include high mortality and, in survivors, significant long-term neurodevelopmental impairment, such as cerebral palsy, epilepsy, cognitive and motor dysfunction, intellectual disability, and sensory deficits (visual and hearing impairments). ABP, arterial blood pressure; CBF, cerebral blood flow; RDS, respiratory distress syndrome; GM, germinal matrix; PDA, patent ductus arteriosus; IVH, intraventricular hemorrhage; PVL, periventricular leukomalacia; PHVD, post-hemorrhagic ventricular dilatation; PHH, post-hemorrhagic hydrocephalus; ROS, reactive oxygen species; F2-isoPs, F2-isoprostanes; AOPPs, advanced oxidation protein products; NPBI, non-protein-bound iron; TH, total hydroperoxide; IL-1β, interleukin-1β; IL-6, interleukin-6; CRP, C-reactive protein; IL-8, interleukin-8; VEGF, vascular endothelial growth factor; Ang2, angiopoietin-2.

The pathogenesis of IVH in preterm neonates is multifactorial, arising from the interplay of vascular immaturity, hemodynamic instability, impaired autoregulatory mechanisms, inflammation, and oxidative stress (6), as illustrated in Figure 1. Preterm infants are particularly susceptible to such hemorrhage due to the persistence of the germinal matrix (GM), a region of the brain characterized by rich but immature vasculature that is fragile and highly vulnerable to impaired cerebral autoregulation and subsequent fluctuations in cerebral blood flow (6–8).

The severity of IVH is assessed according to a classification system based on the extent of bleeding. Mild forms include grade 1 (subependymal hemorrhage limited to the GM) and grade 2 (bleeding into the lateral ventricles without dilatation and/or coagulum affecting less than 50% of the ventricle volume). Severe forms include grade 3 (IVH with ventricular dilatation and/or a coagulum affecting more than 50% of the ventricular volume) and grade 4 (IVH extending into the surrounding parenchyma) (1, 6). Severe IVH is accompanied by neuroinflammation and a marked increase in proinflammatory cytokine production (9, 10).

Cranial ultrasonography is routinely used to detect IVH in preterm infants; however, it only identifies hemorrhage that has already occurred. Because of its often asymptomatic course, bleeding may remain undetected for hours or even days (4). The ability to predict the development of IVH, or at least to detect it early and recognize its complications, paves the way for implementing individualized preventive measures and early therapeutic interventions, thereby helping to prevent irreversible brain injury in preterm infants (1, 4, 8).

### 1.2 Biomarkers

In recent years, considerable research efforts have been directed toward the discovery, characterization, and validation of biomarkers capable of enabling the early prediction of the onset and progression of IVH and other forms of brain injury in newborns (6–8, 11, 12). The integration of reliable biomarkers into routine clinical practice represents a crucial step toward advancing neuroprotection in neonatology (6–8, 12–14). Biomarkers must not only demonstrate high sensitivity and specificity but also allow for rapid and reliable laboratory processing to facilitate the timely implementation of preventive measures in clinical care (3, 8).

Current studies focus on markers of brain injury, inflammatory markers, angiogenic factors, vasoactive substances, growth factors, and indicators of oxidative stress. A promising new direction involves the investigation of genomic, proteomic, and metabolomic markers (6, 10, 11, 15, 16), in which multiple biomarkers are often analyzed in combination (12). Researchers are examining the presence of biomarkers in various tissues and biological fluids (8, 17). In this review, we specifically focus on blood-based biomarkers (cord blood and peripheral blood).

## 2 Particular blood-based biomarkers

### 2.1 Biomarkers of structural brain injury

Biomarkers of structural brain injury (Table 1) are proteins or other molecules commonly found in cells of the central nervous system (CNS), particularly neurons and glial cells. The precise mechanisms by which these biomarkers enter the bloodstream are not yet fully understood. Following brain injury, specific proteins are released into the extracellular space and subsequently into the cerebrospinal fluid (CSF). They may reach the bloodstream via the glymphatic system, active transport, or passive diffusion, particularly in cases of blood–brain barrier (BBB) disruption (18, 19).

**Table 1:** Overview of structural brain injury biomarkers relevant to IVH and other neurological conditions across age groups and biological specimens, and their roles as reported in earlier research.

Marker	Molecular role	Target population	Associations	Severity corr.	Trend	Corr.	Biological specimen	Ref.
Biomarkers of structural brain injury								
S100B	Calcium-binding protein	Adults, children	TBI, stroke, neurodegenerative and psychiatric diseases	Yes	↑	GCS, CT, MRI	CSF, serum/plasma, saliva, urine	(7, 8, 10, 11, 15, 18–22, 26, 64)
		Full-term, preterm neonates and mothers	HIE, IVH, PVL, other brain injuries and complications			CUS, MRI, aEEG	Cord blood, peripheral blood serum/plasma, CSF, urine, saliva, maternal blood, amniotic fluid	
NfL	Cytoskeletal protein	Adults, children	TBI, stroke, MS, other neurodegenerative and psychiatric diseases	Yes	↑	CT, MRI	CSF, serum/plasma	(7, 25, 26, 64)
		Preterm neonates	IVH, PHVD, neurodevelopmental impairment			CUS	CSF, cord blood, peripheral blood serum	
GFAP	Cytoskeletal protein	Adults, children	TBI, stroke, neurodegenerative diseases	Yes	↑	GCS, CT, MRI	Serum, CSF	(7, 12, 15, 18, 26, 27)
		Full-term and preterm neonates	HIE, PWMI, IVH, PHVD, adverse neurological development			CUS, MRI, aEEG	Cord blood, peripheral blood serum, CSF	
UCH-L1	Hydrolytic enzyme	Adults, children	TBI, stroke	Yes	↑	GCS, CT	Serum/plasma, CSF, saliva	(18, 19, 28, 64)
		Late preterm and full-term neonates	HIE			MRI	Cord bood	
CPK-BB	Isoenzyme of creatine kinase	Adults	TBI	Yes	↑	GCS, CT	Serum/plasma, CSF	(7, 8, 30)
		Full-term and preterm neonates	HIE, IVH			CUS	Cord blood, peripheral blood serum	
NSE	Isoenzyme of enolase	Adults, children	TBI, NETs, SCLC, neuroblastoma, stroke, intracerebral hemorrhage	Yes	↑	CT	Serum, CSF, urine	(8, 31, 32, 64)
		Full-term and preterm neonates	Neuroblastoma, HIE, IVH, PVL			CUS	Cord blood, peripheral blood serum, CSF, amniotic fluid	

While the focus is on biomarkers relevant to IVH in preterm neonates, the tables also include findings from studies in adults, term newborns, and older children, as well as analyses of CSF, urine, and other biological samples, to provide a comprehensive overview.

S100B, S100 calcium-binding protein B; NfL, neurofilament light chain; GFAP, glial fibrillary acidic protein; UCH-L1, ubiquitin C-terminal hydrolase L1; CPK-BB, brain-type creatine phosphokinase; NSE, neuron-specific enolase; TBI, traumatic brain injury; HIE, hypoxic–ischemic encephalopathy; IVH, intraventricular hemorrhage; PVL, periventricular leukomalacia; MS, multiple sclerosis; PHVD, post-hemorrhagic ventricular dilatation; PWMI, periventricular white matter injury; NETs, neuroendocrine tumors; SCLC, small cell lung cancer; GCS, Glasgow coma scale; CT, computed tomography; MRI, magnetic resonance imaging; CUS, cranial ultrasound; aEEG, amplitude integrated electroencephalography; CSF, cerebrospinal fluid; ↑, increase, —, not specified.

#### 2.1.1 S100 calcium-binding protein B

S100 calcium-binding protein B (S100B) is a small calcium-binding protein composed of two β-subunits. It is primarily associated with the nervous system and is predominantly produced by astrocytes in response to metabolic stress, although extracerebral sources have also been identified (7, 8, 14, 20, 21). At nanomolar concentrations, S100B promotes neuronal proliferation during development, whereas at micromolar concentrations, it can induce neuronal apoptosis and elicit an inflammatory response within the nervous system (7, 19, 22). According to multiple studies, S100B is regarded as one of the most important early biomarkers for predicting brain damage (including IVH in preterm neonates) and related complications in preterm infants, demonstrating high sensitivity and specificity (7, 8, 12, 20).

#### 2.1.2 Neurofilament light chain

Neurofilaments are essential components of the neuronal cytoskeleton. In the CNS, they consist of three polypeptide chains with distinct molecular weights: neurofilament light chain (NfL), neurofilament medium chain (NfM), and neurofilament heavy chain (NfH), together with the protein *α*-internexin. NfL is the most abundant subunit and is currently recognized as a promising biomarker of neuronal and axonal injury across a broad spectrum of neurological disorders. Neuronal injury, whether acute or chronic, leads to the release of neurofilament fragments into the CSF and subsequently into the systemic circulation (23, 24). NfL reaches its highest concentrations in the CSF, where its levels are several times higher than in peripheral blood. However, with the advent of highly sensitive analytical methods, NfL can now also be reliably detected in serum or plasma (23, 24). Evidence from recent studies supports its importance in preterm infants, showing that elevated NfL concentrations in extremely preterm neonates with IVH correlate with disease severity (23, 25).

#### 2.1.3 Glial fibrillary acidic protein

Glial fibrillary acidic protein (GFAP) is a cytoskeletal protein belonging to the family of intermediate filaments (12, 26). Its primary isoform, GFAP-α, is specific to the CNS, where it is predominantly expressed in astrocytes (18, 26, 27). GFAP is currently under intensive investigation as a biomarker of brain injury in various neuropathological conditions, particularly among adult patients (12, 26, 27). In preterm neonates, however, study results have shown some variability, with several reports emphasizing the need for more sensitive methods to detect GFAP in serum. In neonates with IVH or other forms of brain injury, elevated serum GFAP concentrations have frequently been observed, whereas levels in control groups were often below the detection limit. Consequently, differences between groups were not statistically significant (11, 12).

#### 2.1.4 Ubiquitin C-terminal hydrolase L1

Ubiquitin C-terminal hydrolase L1 (UCH-L1) is a hydrolytic enzyme belonging to the deubiquitinase family that processes ubiquitin, primarily by maintaining its free form within the cell (15, 28, 29). It is highly expressed in neurons and, to a much lesser extent, in the gonads (18, 29). UCH-L1 is currently being investigated as an early, neuron-specific biomarker of brain injury in both pediatric and adult patients with traumatic brain injury, as well as in newborns following perinatal asphyxia leading to hypoxic–ischemic encephalopathy (HIE). It may aid in the early identification of patients at higher risk for adverse neurological outcomes (15, 18, 28, 29). However, its role remains relatively unexplored in preterm infants with IVH.

#### 2.1.5 Brain-type creatine phosphokinase

Brain-type creatine phosphokinase (CPK-BB) is a brain-specific isoenzyme of creatine kinase that catalyzes the conversion of creatine to creatine phosphate using adenosine triphosphate (ATP). Several studies have confirmed its potential as an early biomarker of brain injury (including IVH in preterm infants); however, its clinical utility remains limited due to low specificity and considerable fluctuations in concentration over time (7, 30).

#### 2.1.6 Neuron-specific enolase

Neuron-specific enolase (NSE) is an isoenzyme of enolase composed of two γ-subunits. It is a dimeric protein with a glycolytic function that contributes to energy production in the form of ATP. Under physiological conditions, NSE is predominantly found in tissues of neuroectodermal origin. During the fetal period, it is mainly expressed in the nervous system and immature lung tissue, where it is associated with the activity of pulmonary neuroendocrine cells. In the postnatal period and throughout adulthood, NSE is primarily localized in neurons of the CNS and in peripheral neuroendocrine cells (8, 31, 32).

NSE serves as a biomarker for the diagnosis and monitoring of CNS injury and is also used as a tumor marker for malignancies of neuroectodermal origin, including neuroblastoma in neonates (8, 32). Clinical studies have demonstrated significantly elevated NSE levels in neonates following hypoxic–ischemic insult (8). In preterm infants with IVH, increased concentrations have been observed as late as the third day of life, making NSE a relatively delayed marker of brain injury. Its potential clinical application lies particularly in predicting the development of severe forms of IVH (31).

### 2.2 Biomarkers of oxidative stress

During various pathological and physiological processes, whether endogenous or exogenous, free radicals are produced as by-products of metabolic redox reactions. These highly unstable molecules with unpaired electrons, including reactive oxygen and nitrogen species, must be tightly regulated and maintained at low levels. Free radicals play important roles in several cellular processes, such as signaling pathways and immune defense against pathogens. However, their excessive accumulation in the body can be harmful (33).

When the balance between the production of free radicals and the cell's ability to eliminate them is disrupted, the antioxidant capacity becomes overwhelmed, leading to the overproduction of free radicals and the development of oxidative stress. This process damages essential cellular structures such as lipids, proteins, polysaccharides, and deoxyribonucleic acids (8, 33, 34). The induction of antioxidant enzymes, such as superoxide dismutase, catalase, and glutathione peroxidase, becomes significantly more pronounced only during the third trimester of pregnancy. These enzymes provide protection against oxidative stress during the transition from the intrauterine to the extrauterine environment. After birth, the newborn transitions from a relatively hypoxic intrauterine state to an environment with a higher partial pressure of oxygen, creating conditions for increased production of oxygen radicals. Extremely premature newborns are particularly exposed to heightened oxidative stress due to underdeveloped antioxidant defenses. Oxidative stress can significantly contribute to the development of several serious conditions, including respiratory distress syndrome (RDS), bronchopulmonary dysplasia (BPD), necrotizing enterocolitis (NEC), patent ductus arteriosus (PDA), and, not least, intraventricular hemorrhage (IVH) and diffuse white matter injury. In cases of impaired postnatal adaptation, when oxygen resuscitation, artificial pulmonary ventilation, and other intensive care interventions are required, oxidative stress increases further, contributing to additional tissue damage, including brain injury (Figure 1) (8, 33, 34). Specific molecules involved in oxidative stress may therefore serve as early biomarkers of free radical–associated diseases in preterm infants (Table 2) (8, 34). In preterm neonates who develop mild to severe IVH, higher concentrations of oxidative stress markers, including advanced oxidation protein products (AOPPs), total hydroperoxides (TH), and non–protein-bound iron (NPBI), have been detected in cord blood (34).

**Table 2:** Overview of oxidative stress and inflammatory biomarkers relevant to IVH and other types of brain damage across age groups and biological specimens, based on previous research.

Marker	Molecular role	Target population	Associations	Severity corr.	Trend	Corr.	Biological specimen	*Ref.*
Biomarkers of oxidative stress								
AOPPs, TH, NPBI	Specific molecules involved in oxidative stress	Full-term and preterm neonates	HIE, IVH, PVL and complications	Yes	↑	CUS, MRI	Cord blood, peripheral blood plasma, CSF	(8, 26, 34, 65)
F2-isoPs	Bioactive compounds belonging to the eicosanoid family	Full-term and preterm neonates	Brain injury, incl. PVL and IVH, neurodevelopmental impairment	Yes	↑	MRI, aEEG	Cord blood, peripheral blood plasma, CSF	(8, 35, 65)
UA	Degradation product of purine bases	Adults	Stroke	—	↑	CT	Peripheral blood serum, CSF	(7, 26)
		Preterm neonates	IVH, PVL			CUS	Peripheral blood serum/plasma, CSF	
24S-HC	Product of the enzymatic oxidation of cholesterol	Adults	TBI, neurodegenerative diseases	—	↑	CT, MRI	Peripheral blood serum/plasma	(8)
		Animal neonatal models	HIE	Yes		—		
COHb	Complex of hemoglobin with carbon monoxide	Full-term and preterm neonates	HIE, grade 2–4 IVH	Yes	↑	CUS, MRI	Arterial or capillary blood gas samples	(38)
EPO	Glycoprotein hormone	Preterm neonates	HIE, IVH, other brain injuries and complications	Yes	↑	CUS	Cord blood, amniotic fluid	(7, 11, 26, 39)
Inflammatory markers								
CRP	Acute-phase protein	Adults	Stroke	Yes	↑	CT	Serum	(7, 10, 26, 44)
		Preterm neonates and mothers	IVH	—		CUS	Cord blood, peripheral blood serum, maternal serum	
IL-6	Proinflammatory cytokine	Adults	Stroke	—	↑	CT	Serum	(7, 8, 10, 20, 26, 44)
		Full-term and preterm neonates and mothers	HIE, IVH, PVL, other brain injuries, adverse neurological development			CUS, MRI, aEEG	Cord blood, peripheral blood serum, CSF, maternal serum, amniotic fluid	
IL-1β, IL-8, TNF-α	Proinflammatory cytokines	Adults	TBI, stroke	—	↑	CT	Serum, CSF	(8, 10, 20)
		Full-term and preterm neonates	HIE, IVH, PVL, other brain injuries			CUS, MRI, aEEG	Cord blood, peripheral blood serum, amniotic fluid, CSF	
CCL18	Chemotactic cytokine	Preterm neonates	Grade 2–4 IVH, cerebral palsy	—	↓	CUS	Cord blood, peripheral blood plasma	(7, 26, 44)

AOPPs, advanced oxidation protein products; TH, total hydroperoxide; NPBI, non-protein-bound iron; F2-isoPs, F2-isoprostanes; UA, uric acid; 24S-HC, 24S-hydroxycholesterol; COHb, carboxyhemoglobin; EPO, erythropoietin; CRP, C-reactive protein; IL-6, interleukin-6; IL-1β, interleukin-1β; IL-8, interleukin-8; TNF-α, tumor necrosis factor α; CCL18, CC chemokine ligand 18; HIE, hypoxic–ischemic encephalopathy; IVH, intraventricular hemorrhage; PVL, periventricular leukomalacia; TBI, traumatic brain injury; CUS, cranial ultrasound; MRI, magnetic resonance imaging; aEEG, amplitude integrated electroencephalography; CT, computed tomography; CSF, cerebrospinal fluid; ↑, increase; ↓, decrease; —, not specified.

#### 2.2.1 F2-Isoprostanes

F2-isoprostanes (F2-isoPs) are bioactive compounds belonging to the eicosanoid family, known for their vasoconstrictive effects, particularly in the context of oxidative stress. Unlike classical eicosanoids, they are formed through non-enzymatic lipid peroxidation in cell membranes, where free radicals react with arachidonic acid without the involvement of cyclooxygenase enzymes (8, 35). Current research suggests that F2-isoPs may serve as early biomarkers of oxidative stress, especially in newborns at increased risk for brain injury. Elevated plasma levels of F2-isoPs have been detected immediately after birth in preterm infants who later developed periventricular leukomalacia (PVL), severe forms of IVH, and neurodevelopmental impairments (8, 34, 35).

#### 2.2.2 Uric acid

Uric acid (UA) is formed through the degradation of purine bases (7). The intermediate compound hypoxanthine accumulates at sites of brain injury caused by hypoxia and is converted to uric acid by the enzyme xanthine oxidase during reperfusion. This reaction also generates reactive oxygen species that may contribute to further ischemia–reperfusion injury. Uric acid has been investigated as a potential predictive biomarker in patients with stroke and in preterm neonates with IVH, but study results have been inconsistent (7, 26).

#### 2.2.3 24S-hydroxycholesterol

24S-hydroxycholesterol (24S-HC) is a product of the enzymatic oxidation of cholesterol, which is abundant in the brain and often referred to as “cerebrosterol.” This metabolite is synthesized exclusively in the brain by the neuron-specific cytochrome P450 enzyme cholesterol 24-hydroxylase. 24S-HC can cross the BBB and bind to lipoproteins in the peripheral circulation, where it is subsequently converted into bile acids and excreted in the bile (8, 36). In adults, 24S-HC is well established as a biomarker of neurodegenerative diseases and traumatic brain injury. Although direct clinical studies in human neonates are still lacking, preclinical animal studies suggest its potential as a biomarker of hypoxic–ischemic brain injury and as an indicator of the severity of such injury (8, 36). Additional research is required to determine the specific role of 24S-HC in IVH.

#### 2.2.4 Carboxyhemoglobin

Carboxyhemoglobin (COHb) is a complex of hemoglobin with carbon monoxide (CO) that is formed primarily in erythrocytes, either as a result of exogenous CO inhalation or endogenous CO production via heme catabolism by the enzyme heme oxygenase (mainly HO-1). Under physiological conditions, erythrocytes are the principal site of its formation; however, under pathological conditions such as hemolysis, COHb can also form in plasma from cell-free hemoglobin (37). The activity of HO-1, and consequently endogenous CO production, increases in response to oxidative stress and inflammation; therefore, COHb levels can be considered an indirect biomarker of these processes (37, 38). Elevated COHb levels have been reported in preterm neonates and have been associated with an increased risk of grade 2–4 IVH (38).

#### 2.2.5 Erythropoietin

Erythropoietin (EPO) is a glycoprotein hormone primarily synthesized in the kidneys in response to oxygen deprivation at the cellular and tissue levels. Although it is mainly involved in the regulation of erythropoiesis, several studies have suggested that EPO may also exert neuroprotective effects. Elevated EPO levels have been detected in the cord blood of preterm infants with IVH or other forms of brain injury (7, 8, 39, 40).

### 2.3 Inflammatory markers

**Proinflammatory cytokines** that serve as key mediators of the inflammatory response include *interleukin-6 (IL-6), interleukin-8 (IL-8), interleukin-1*β *(IL-1*β*), and tumor necrosis factor-alpha (TNF-*α*).* These cytokines are produced by various cell types, particularly those of the immune system, in response to tissue damage or the presence of infectious agents. Their primary function is to initiate and amplify the inflammatory response, including the recruitment of other immune cells to the site of injury (10, 41). Findings regarding inflammatory markers associated with IVH and other types of brain injury are summarized in Table 2.

#### 2.3.1 C-reactive protein and interleukin-6

C-reactive protein (CRP) is a major acute-phase protein involved in inflammation. It is primarily synthesized by hepatocytes in response to IL-6, and its serum concentration increases significantly in patients with various inflammatory conditions, most commonly bacterial infection, ischemia, malignancy, or traumatic injury (10, 42). In addition to its role as a diagnostic marker, CRP is actively involved in modulating inflammatory processes, including those affecting the vascular wall, thereby contributing to the development of endothelial dysfunction and vascular pathology (10).

Hypoxic–ischemic brain injury activates microglia, which subsequently produce proinflammatory cytokines and other mediators. These substances promote neuroinflammation and can lead to secondary damage to neural tissue (8). In adult stroke patients, elevated CRP levels during the acute phase of the disease have been correlated with the extent of brain damage, with higher CRP levels being associated with an unfavorable prognosis (10). In premature newborns, numerous studies have investigated the role of proinflammatory cytokines as potential early biomarkers of adverse neurological outcomes (26). Some studies have also demonstrated an association between elevated maternal serum IL-6 and CRP levels and an increased risk of IVH in preterm infants. Although these biomarkers have shown some predictive potential, their clinical utility in detecting IVH or other brain injuries remains limited due to their nonspecific nature (7).

#### 2.3.2 CC chemokine ligand 18

CC chemokine ligand 18 (CCL18) is a chemotactic cytokine involved in the immune response (7, 43). According to available data, elevated levels of CCL18 may exert a protective effect against IVH and other forms of brain injury. This is related to the immunohistochemically demonstrated presence of the CCR3 receptor in the germinal matrix, ependyma, and choroid plexus, where CCL18 acts as its functional antagonist (7, 8, 44).

### 2.4 Growth factors and vasoactive substances

An overview of growth factors and vasoactive substances involved in IVH and other forms of brain injury is provided in Table 3.

**Table 3:** Growth factors, vasoactive substances, and molecular biomarkers associated with IVH and related brain injuries, as reported in previous research.

Biomarker	Molecular role	Target population	Associations	Severity corr.	Trend	Corr.	Biological specimen	Ref.
Growth factors and vasoactive substances								
Activin A	Growth factor	Full-term and preterm neonates	HIE, IVH, PVL, other brain injuries	Yes	↑	CUS, MRI	CSF, peripheral blood serum/plasma, cord blood, amniotic fluid, urine, saliva	(5, 7, 15, 22, 26, 45)
GDF15	Regulatory protein	Preterm neonates	Severe IVH	—	↑	CUS	Peripheral blood serum	(46)
TGF-β1	Regulatory protein	Preterm neonates	PHVD, WMI	Yes	↑	CUS, MRI	CSF	(7, 26)
AM	Vasoactive peptide hormone	Children	TBI	Yes	↑	CT	CSF	(15, 22, 26)
		Full-term and preterm neonates	IVH, HIE			CUS	Cord blood, peripheral blood plasma, urine, saliva	
Copeptin	Glycopeptide hormone	Adults	Stroke	—	↑	CT	Serum	(49)
		Full-term and preterm neonates	Perinatal asphyxia, IVH			—	Peripheral blood plasma	
Genomics, transcriptomics, proteomics, and metabolomics								
mRNA, miRNA, lncRNA	RNA molecules	Animal models, adults, children	TBI, stroke, brain tumors, MS, other neurodegenerative diseases	—	↑	CT, MRI	CSF, serum/plasma, saliva	(9, 52, 64, 66)
		Preterm neonates	IVH			CUS	Peripheral blood serum/plasma, CSF	
Alanine, aspartate, glutamate, ornithine	Aminoacids	Adults	Stroke, TBI	—	-	CT	Serum/plasma	(16)
		Full-term and preterm neonates	HIE, IVH		↑	MRI, CUS	Cord blood, peripheral blood serum, urine	
PGF, LAIR-2, TRAIL-R2, BOC	Proteins	Preterm neonates	IVH	—	↑	CUS	Cord blood	(3, 54)
PCs	Glycerophospho-lipids	Preterm neonates	IVH	Yes	↓	CUS	Cord blood	(3, 54)

GDF15, growth differentiation factor 15; TGF-β1, transforming growth factor beta 1; AM, adrenomedullin; mRNA, messenger RNA; miRNA, microRNA; lncRNA, long non-coding RNA; PGF, placental growth factor; LAIR-2, leukocyte-associated immunoglobulin-like receptor 2; TRAIL-R2, tumor necrosis factor-related apoptosis-inducing ligand receptor 2; BOC, brother of CDO; PCs, phospatidylcholines; HIE, hypoxic–ischemic encephalopathy; IVH, intraventricular hemorrhage; PVL, periventricular leukomalacia; PHVD, post-hemorrhagic ventricular dilatation; WMI, white matter injury; TBI, traumatic brain injury; MS, multiple sclerosis; CUS, cranial ultrasound; MRI, magnetic resonance imaging; CT, computed tomography; CSF, cerebrospinal fluid; ↑, increase; ↓, decrease; —, not specified.

#### 2.4.1 Activin A

Activin A is a growth factor, a protein composed of two identical β subunits linked by disulfide bonds (5, 7, 22). It is involved in regulating cell proliferation and differentiation, including that of neuronal cells, and plays an important role in the activation of neuroprotective mechanisms following acute brain injury (5, 7, 14, 22, 45). Several studies have reported significantly higher serum levels of activin A in preterm infants with acute brain injury (hemorrhagic, hypoxic–ischemic, or infectious) compared with controls without such injury. These levels also reflected the severity of neurological impairment (5, 7, 14, 22, 39).

#### 2.4.2 Growth and differentiation factor 15

Growth and differentiation factor 15 (GDF15) is a member of a group of regulatory proteins known as the Transforming Growth Factor Beta (TGF-β) superfamily, which also includes TGF-β1. GDF15 is a cytokine whose expression increases in response to various pathological conditions, including inflammation, oxidative stress, hypoxia, ischemia, and tissue damage (46, 47). In preterm infants, an association has been demonstrated between elevated early levels of GDF15 and adverse outcomes, including severe IVH. This relationship has been attributed to a systemic inflammatory and stress response, with a possible direct role of GDF15 in affecting platelet integrity (46). TGF-β1 is a cytokine that has been studied as a biomarker for the development of complications associated with IVH, but it is primarily measured in CSF (26).

#### 2.4.3 Adrenomedullin

Adrenomedullin (AM) is a 52-amino-acid vasoactive peptide hormone that is upregulated in response to hypoxia and inflammation. It is involved in the regulation of blood pressure and blood flow, promotes pulmonary vasodilation and bronchodilation, stimulates angiogenesis and lymphangiogenesis, exerts anti-inflammatory effects, and participates in a wide range of other physiological processes (22, 26, 48). It also plays a significant role in the CNS, where it helps regulate cerebral blood flow. Some studies have shown that elevated AM levels measured shortly after birth are associated with the subsequent development of IVH in preterm infants and correlated with its severity (22).

#### 2.4.4 Copeptin

Copeptin is a glycopeptide composed of 39 amino acids and represents the stable C-terminal fragment of the vasopressin precursor (49). Because it is released in equimolar ratio with vasopressin, its level can serve as an indirect indicator of vasopressin secretion, which increases in response to hemodynamic, osmotic, and stress stimuli (10, 49). Copeptin has been studied mainly in adult patients as a predictive and diagnostic marker in critical conditions, including spontaneous intracerebral hemorrhage (10, 49). Data on the relationship between copeptin and brain injury in preterm neonates remain limited. However, existing studies suggest that elevated copeptin levels are associated with perinatal distress and poorer neurodevelopmental outcomes (50).

### 2.5 Genomics, transcriptomics, proteomics, and metabolomics

The multi-omic approach, that is, the study of the genome, transcriptome, proteome, and metabolome, represents a modern tool for identifying potential biomarkers of IVH in premature newborns (Table 3) (51). Transcriptomic studies have revealed changes in the expression of messenger RNA (mRNA), microRNA (miRNA), and long non-coding RNA (lncRNA) in the peripheral blood of preterm newborns with IVH. Notably, the lncRNA H19 has been identified as potentially significant. Functional analyses suggest associations between altered RNA expression and disorders of heme metabolism, ferroptosis, interferon response, and lymphocyte activation. LncRNAs are defined as transcripts longer than 200 nucleotides and are involved in regulating a number of cellular processes, including the development of neurons and immune cells. Their dysregulation has been associated with neurological, oncological, and immunological diseases (52, 53).

Metabolomic studies focusing on amino acid and carnitine profiling have shown increased levels of **alanine, aspartate, glutamate, and ornithine** immediately after birth in preterm neonates who subsequently developed IVH. These changes are attributed to impaired amino acid metabolism caused by hypoxia and ischemia, reduced energy production, and decreased neurotransmitter synthesis, all of which may contribute to brain damage. **Carnitine** plays a crucial role in transporting fatty acids from the cytosol to the mitochondria for energy production. In particular, the acylcarnitines C5-OH and C14:1 have been identified as potentially important in neonates with IVH (16).

Proteomic–metabolomic analyses have further revealed increased expression of **placental growth factor** (PGF), **leukocyte-associated immunoglobulin-like receptor 2** (LAIR-2), **tumor necrosis factor–related apoptosis-inducing ligand receptor 2** (TRAIL-R2), and **the cell adhesion protein BOC** (Brother of CDO). Concurrently, decreased levels of specific **phosphatidylcholines** have been observed, which may indicate disruption of the BBB (3, 54).

#### 2.5.1 Placental growth factor

Placental growth factor (PGF) is an angiogenic factor belonging to the vascular endothelial growth factor (VEGF) family, which plays a central role in the formation, growth, and remodeling of blood vessels. PGF is predominantly produced by the placenta, where it contributes to the growth and differentiation of trophoblast cells (54, 55). Balanced signaling between VEGF and PGF is particularly important during the development of the fetal vascular system, including the cerebral vasculature. Disruption of this balance in the germinal matrix, which is especially fragile in preterm infants, has been suggested as a potential contributing factor to the increased risk of IVH (54).

#### 2.5.2 Leukocyte-associated immunoglobulin-like receptor 2

Leukocyte-associated immunoglobulin-like receptor 2 (LAIR-2) is a soluble protein belonging to the immunoglobulin superfamily that binds to collagen. It is primarily secreted by hematopoietic cells. LAIR-2 acts as a competitive antagonist of the membrane receptor LAIR-1, thereby modulating its inhibitory signaling. Through this mechanism, it participates in the regulation of inflammatory responses, promotes angiogenesis, and may contribute to placental implantation. By interacting with collagen, LAIR-2 may also indirectly influence collagen-induced platelet adhesion and aggregation (54, 56).

#### 2.5.3 Tumor necrosis factor–related apoptosis-inducing ligand receptor 2

Tumor necrosis factor–related apoptosis-inducing ligand receptor 2 (TRAIL-R2), also known as DR5, is a receptor belonging to the TNF receptor superfamily. It is expressed on the surface of various cell types, including cells of the monocyte–macrophage system, endothelial cells, and vascular smooth muscle cells. Upon binding to its ligand TRAIL, the receptor can activate apoptotic signaling pathways and is also involved in regulating cell survival and angiogenesis, depending on the cellular context. Increased expression of TRAIL-R2 has been observed in certain tumor-associated endothelial cells, where it may contribute to altered vascular integrity and potentially to bleeding. In premature newborns, a possible association has been suggested between elevated TRAIL-R2 expression and impaired development of the cerebral vascular network, which may play a role in the pathogenesis of IVH (54, 57).

#### 2.5.4 Brother of CDO

Brother of CDO (BOC) is a cell surface receptor belonging to the immunoglobulin superfamily. It functions as a co-receptor in the Sonic hedgehog (Shh) signaling pathway and plays a key role in embryonic development and proper tissue organization, particularly in neuronal development, muscle differentiation, and vascular formation. Recent studies suggest a link between disruption of BOC regulation and Shh signaling in the development and progression of IVH, while altered Shh signaling in endothelial cells may lead to disruption of the BBB (54, 58).

## 3 Laboratory challenges

A wide range of laboratory techniques is currently available for the analysis of biomarkers relevant to neonatal care. Within immunochemistry, several methodologies are used in practice, including chemiluminescence immunoassay (CLIA), chemiluminescent microparticle immunoassay (CMIA), electrochemiluminescence immunoassay (ECLIA), and fluorescent immunoassay (FIA); time-resolved fluorescence immunoassay (TRFIA) is applied less frequently, while radioimmunoassay (RIA) has largely been replaced by nonradioactive methods. In routine diagnostics, automated techniques are most widely implemented for clinically relevant biomarkers such as IL-6 and CRP (14, 59). Their advantages include automation, reproducibility, rapid turnaround time, and compatibility with high-throughput laboratory systems. In contrast, the enzyme-linked immunosorbent assay (ELISA) remains primarily a research tool, offering flexibility for the quantification of less commonly available analytes such as cytokine panels, GFAP, or neurofilament light chain (NfL) (15, 31). Chromatographic techniques, including high-performance liquid chromatography (HPLC) and ultrahigh-performance liquid chromatography with tandem mass spectrometry (UHPLC-MS/MS), are essential for metabolic screening. Mass spectrometry, often combined with chromatographic separation, remains the gold standard for metabolomic studies and provides high specificity and sensitivity. Additionally, molecular biology techniques such as polymerase chain reaction (PCR) and RNA sequencing are used to analyze nucleic acids and transcriptome profiles (52, 59). Multiplex approaches can enable the simultaneous quantification of tens to hundreds of proteins from minimal sample volumes, including central nervous system–specific markers (17, 24).

The translation of newly identified biomarkers into clinical practice requires both analytical and clinical validation. Critical steps include establishing analytical and clinical sensitivity and specificity, defining reference intervals, and ensuring interlaboratory standardization supported by external quality control. Stability studies of biological samples during storage and transport are essential, as is the evaluation of potential interferences. Furthermore, integrating economic evaluations is increasingly recognized as necessary to assess the feasibility and long-term sustainability of new biomarker assays in routine care (15, 24).

Despite substantial progress in biomarker discovery, several barriers to implementation remain. Access to advanced analytical platforms is often limited to specialized research centers, restricting broader clinical adoption. Many techniques require highly trained personnel and structured quality assurance systems, which may not be available in all neonatal intensive care settings. Incorporating new assays into established hospital workflows and electronic health record systems can also be technically and financially challenging. Collectively, these obstacles slow the translation of promising biomarker candidates from the experimental phase to widespread clinical use. At the same time, emerging directions offer new opportunities. The role of point-of-care testing (POCT) is steadily expanding. Portable and bedside devices now enable rapid measurement of selected biomarkers, such as CRP, directly in the intensive care unit. This advancement has the potential to shorten turnaround times, accelerate clinical decision-making, and facilitate earlier initiation of neuroprotective strategies (6, 8).

In parallel, machine learning (ML) and artificial intelligence (AI) are increasingly being applied to the large and complex datasets generated by proteomic, metabolomic, and transcriptomic approaches. These computational methods support pattern recognition, multidimensional data integration, and the development of predictive diagnostic and prognostic models. Laboratory diagnostics is progressively incorporating such methodologies, and the integration of ML into biomarker research represents a promising pathway toward more precise, individualized, and clinically relevant neonatal care (51, 60).

## 4 Discussion

Current research indicates strong potential for various biomarkers in both predicting the onset of IVH and monitoring its progression. Biomarkers such as S100B, Activin A, and NfL, among others, have repeatedly shown promise, particularly when used in combination with other biomarkers or diagnostic strategies. However, many existing studies are limited by methodological shortcomings. These often include small and heterogeneous patient cohorts, variability in biomarker analysis methods, inconsistent timing of sample collection, and failure to account for potentially confounding factors such as perinatal asphyxia, sepsis, or other perinatal conditions (14, 15). Further complicating research efforts is the significant variability of neurological complications occurring during the perinatal period. As a result, many biomarkers are also being investigated in relation to other types of brain injury or complications associated with preterm birth. Nevertheless, this does not preclude their potential applicability in the context of IVH.

The pathogenetic mechanisms of brain injury partially overlap across different age groups, as reflected by the occurrence of shared biomarkers (Tables 1–3). Biomarkers are currently being extensively studied in various neurological disorders, particularly in adult patients, whereas data concerning the pediatric population, and especially newborns, remain limited. Under physiological conditions, some biomarkers are present in the CSF and blood only at very low concentrations, and disruption of the BBB may lead to their elevation (25).

In traumatic brain injury, multiple pathophysiological processes are involved, reflecting the heterogeneity of the injury itself. The initial, or primary, phase occurs at the moment of impact, when mechanical forces directly damage neural tissue and compromise the BBB. This is followed by a secondary phase, characterized by widespread neuroinflammatory and systemic immune responses. During this stage, immune cells release cytokines, adhesion molecules, and growth factors that activate multiple interacting pathways. Over the course of hours to months, these mechanisms can either support tissue repair or contribute to metabolic imbalance and oxygen deprivation resulting from cerebral edema (19). In these patients, markers of structural brain injury are particularly useful; these are proteins that are physiologically present in the CNS and released following neural cell damage and BBB disruption. In term newborns, brain injury is most commonly caused by HIE, neonatal stroke, or infections, which can affect neural stem cells during a critical period of brain development (8).

For pediatric patients, specific reference values are essential; however, such data are often lacking, particularly for newborns and preterm infants. In preterm neonates, unique pathogenetic mechanisms are involved, including the presence of the germinal matrix, overall immaturity of the organism (including the antioxidant defense system), and the high vulnerability of the developing brain (61). In these infants, biomarker concentrations must be interpreted in relation to the degree of immaturity. Biomarker levels often correlate inversely with gestational age and body weight, increase during the first days of life, and are associated with factors related to brain injury. Preterm birth is associated with increased brain vulnerability; the ongoing intensive neuronal turnover and elevated biomarker levels may reflect a neuronal stress response to birth (25, 62).

To ensure sufficient predictive value, it is essential that biomarker sampling be performed as early as possible after birth, with results available on the same day, ideally at the bedside. Such an approach would enable the early identification of neonates at increased risk for developing IVH. There is an ongoing need to identify novel biomarkers and to validate existing ones in larger, ideally multicenter, studies involving broader cohorts of preterm neonates. It is crucial to eliminate potentially confounding factors in order to integrate the most promising biomarkers into routine clinical practice within neonatal intensive care units.

Further research is required before predictive biomarkers can be implemented in standard clinical use, including the establishment of reference ranges specific to the population of preterm neonates. Additionally, monitoring dynamic changes in biomarker levels through repeated sampling during the first days of life is important, as it enables the assessment of not only IVH onset risk but also its progression. Current research strategies aim to uncover the complex pathophysiological mechanisms underlying various disease processes, including IVH. These approaches lay the foundation for a deeper understanding of IVH pathogenesis, the discovery of new biomarkers, and the advancement of neuroprotective approaches (51, 63).

Early identification of neonates at increased risk of developing IVH represents a critical step toward individualized monitoring, the implementation of targeted interventions, and the provision of personalized care. These measures have the potential to significantly improve clinical outcomes and reduce the incidence of long-term neurological complications in preterm infants.
